# Role of serial lactate measurement to predict 28-day mortality in patients undergoing emergency laparotomy for perforation peritonitis: prospective observational study

**DOI:** 10.1186/s40560-019-0418-9

**Published:** 2019-12-11

**Authors:** S. P. Jobin, Souvik Maitra, Dalim Kumar Baidya, Rajeshwari Subramaniam, Ganga Prasad, Vathulru Seenu

**Affiliations:** Department of Anaesthesiology, Pain Medicine and Critical Care, All India Institute of Medical Sciences, Room No 5011, 5th Floor, Teaching Block, New Delhi, 110029 India

**Keywords:** Lactate, Perforation peritonitis, Emergency laparotomy

## Abstract

**Background:**

Serial lactate measurement is found to predict mortality in septic shock. Majority of patients with perforation peritonitis for emergency laparotomy are in sepsis and mortality rate is substantial. However, lactate dynamics has not been studied in this patient population.

**Methods:**

After institutional ethics clearance and informed written consent, 113 patients with suspected or proven perforation peritonitis presenting for emergency laparotomy were recruited in this prospective observational trial. Baseline Mannheim peritonitis index (MPI), SOFA and APACHE II score were calculated. Lactate values were obtained at baseline, immediate and 24-h postoperative period. Primary outcome was 28-day mortality.

**Results:**

Mortality was 15.04% at 28 days. Age, SOFA, qSOFA, APACHE, preoperative lactate, MPI and site of perforation were significantly different between survivors and non-survivors. Arterial lactate values at preoperative (cut off 2.75 mmol/L), immediate postoperative (cut off 2.8 mmol/L) and 24 h-postoperative period (cut off 2.45 mmol/L) independently predicted mortality at day 28. Combination of MPI and 24-h lactate value was best predictor of mortality with AUC 0.99.

**Conclusion:**

Preoperative, immediate postoperative and 24-h postoperative lactate value independently predict 28-day mortality in perforation peritonitis patients undergoing emergency laparotomy. Combination of MPI and 24-h lactate value is the most accurate predictor of mortality.

**Trial registration:**

Clinical Trial Registry of India - CTRI/2018/01/011103

## Background

Patients undergoing emergency laparotomy for intra-abdominal infection experience high peri-operative mortality despite of advancement of surgical technique, antibiotic therapy and intensive care support. In the UK, overall 30-day mortality is one in seven and reaches almost one in four for the elderly (age ≥ 80 years) [1]. Although majority of patients are elderly, with significant comorbidity and high-risk insults such as sepsis, evidence suggests that intervention can improve outcomes. Data from India reveal an overall mortality of 17.86% in these patients [2]. Acute secondary peritonitis represents the common presentation of gastro-intestinal perforation and can be either chemical or bacterial [3]. Perforation peritonitis is linked with multiple organ dysfunction syndrome (MODS) in up to 73% patients and mortality increases up to 30% in such cases [4, 5]. Early identification of patients with higher risk of mortality will be helpful to institute adequate resuscitation and intensive care management [6].

Serum lactate has been studied for its diagnostic and prognostic utility even at early phases of sepsis in critically ill patients. A lactate value > 4 mmol/L in sepsis or septic shock indicates the need for aggressive resuscitation [7]. A prospective observational study conducted in 94 patients in surgical intensive care unit (ICU) admitted with severe sepsis or septic shock found that lactate clearance both at 6 h and 24 h were higher in survivors than in non survivors. Twenty-four-hour lactate clearance was the best independent predictor of 28-day mortality in such patients [8]. Another retrospective study reported that baseline lactate of 2.5 mmol/l or more was a predictor of 28-day mortality in patients with sepsis or septic shock [9].

However, prognostic utility of serial measurement of lactate value has not been specifically studied the subset of sepsis population who present with secondary peritonitis undergoing emergency laparotomy [10]. Therefore, we planned this prospective observational study to identify the usefulness of preoperative baseline lactate and serial measurement of lactate till 24 h in the setting of patients undergoing emergency laparotomy for suspected or proven perforation peritonitis. We hypothesised that serial lactate value up to 24 h will be able to predict 28-day mortality in this group of patients.

## Methods

After obtaining institute ethics committee approval and written informed consent from participants or their legally acceptable representatives, *n* = 113 patients were recruited in this prospective observational study between January 2018 and August 2019. The study was registered in the National Clinical Trial Registry of India (www.ctri.nic.in CTRI/2018/01/011103). Adult patients of either sex aged between 18 and 65 years and American Society of Anaesthesiologists Physical Status (ASA PS) I or II, with proven or clinically suspected peritonitis undergoing emergency laparotomy under general anaesthesia were recruited in this study. Patients or relatives who refused to provide consent, had underwent prior exploratory laparotomy in the current hospitalisation period, had history of ICU stay in last 6 months, had pre-existing significant cardiac, renal and hepatic diseases, chronic alcohol intake and intra-operative massive blood loss or massive transfusion were excluded.

A detailed history of the patient’s current illness, previous history of surgery, drug allergy, other comorbid illnesses (if present, treatment they are taking and the severity of the comorbidity) were recorded along with baseline vitals. Airway examination and systematic examination of the patient during the preanesthetic evaluation of the patient were carried out. Preoperative investigations including complete hemogram, serum electrolytes, renal function test and coagulation parameters were recorded as per standard Institute protocol. Mannheim Peritonitis Index (MPI) [11], Sequential Organ Failure Assessment score (SOFA) and Acute Physiology And Chronic Health Evaluation II (APACHE II) were calculated for each patients. Intra-operative monitoring consisted of electrocardiogram (ECG), oxyhemoglobin saturation (SpO_2_), capnogram (EtCO_2_), non-invasive and invasive blood pressure (IBP) and temperature. Under local anaesthesia, radial artery was cannulated, and preoperative arterial blood gas analysis (Stat Profile® pHOx® Ultra by Nova Biomedical) was done to establish preoperative baseline lactate.

The standard anaesthesia management protocol was followed. General anaesthesia was induced with fentanyl 2 mcg/kg, induction agent (propofol 2–3 mg/kg or ketamine 2 mg/kg or etomidate 0.3 mg/kg) and muscle relaxant (atracurium 0.5 mg/kg or rocuronium 0.9 mg/kg or succinylcholine 2 mg/kg). Volume control ventilation (tidal volume of 6–8 ml/kg, frequency 12–14/min and positive end-expiratory pressure 5–8 cm H_2_O) was used targeting EtCO_2_ of 35–40 mmHg.

Anaesthesia was maintained with isoflurane (end tidal concentration 1–1.2%) in O_2_ and air with FiO_2_ of 0.5 and intermittent fentanyl 0.5–1 μg/kg. Central venous catheter (CVC) was inserted in patients who required vasopressor support for the maintenance of the mean arterial blood pressure (MAP) > 65 mmHg or who were planned for parenteral nutrition in advance.

Patients who were hemodynamically stable and able to maintain normoxia and normocarbia on spontaneous breathing were extubated and transferred to the Post Anaesthesia Care Unit (PACU) and received oxygen by facemask @ 5 L/min. Patients who required postoperative ventilatory support or vasopressor support were managed in the ICU or high dependency unit (HDU) as per standard protocol of the institute. Balanced salt solution was used for fluid resuscitation, and noradrenaline was the vasopressor of choice whenever required in the intraoperative and postoperative period. Fluid and vasopressor management was guided by invasive arterial, central venous pressure, blood gas with lactate and point of care ultrasound monitoring. Broad spectrum antibiotics were initiated at presentation as per institute protocol and appropriate cultures (blood, urine, abdominal fluid and tracheal aspirate whenever suitable) were sent.

### Data collection

At the time of preoperative evaluation, Sequential Organ Failure Assessment score (SOFA), quick-SOFA (qSOFA) and Acute Physiology and Chronic Health Evaluation II (APACHE II) were calculated. After surgery, Mannheim Peritonitis Index (MPI) was also calculated. Arterial lactate and ScVO_2_ (when CVC was in-situ) values were obtained preoperatively, after surgery and at 24 h after surgery.

Patients were followed up daily till 28 days or death or discharge from the hospital, whichever was earlier. Following parameters were collected daily for all patients: urine output, serum creatinine, requirement of renal replacement therapy, development of acute respiratory failure, need for mechanical ventilation, vasopressor requirement, type of nutritional support, length of postoperative ICU stay and length of postoperative hospital stay.

Renal failure was defined as per Kidney Disease Improving Global Outcome (KDIGO) criteria [12]. Circulatory failure was defined as the inability to maintain mean arterial pressure more than 65 mmHg without vasopressor support. Respiratory failure was defined as the PaO_2_/FiO_2_ < 300 and/or the requirement of non-invasive/mechanical ventilation to maintain oxygenation.

### Sample size calculation and statistical analysis

Approximately 10–12 patients undergo emergency laparotomy for perforation peritonitis in our hospital each month. Considering a study period of 15–18 months, we assumed that around 180 patients would be operated in this period. A previous observational study from India reported that mortality in such patients is 17.8% [2]. Considering a finite population of 180, hypothesised % of 28-day mortality in that population is 17.8% ± 5 and a confidence interval of 95%, at least 101 patients was required in this study [13]. With an expected dropout of 10%, *n* = 113 patients were recruited.

All collected data were tabulated in the Microsoft Excel™ [Microsoft Corp., Redmond, WA] Data were presented as median and inter-quartile range (IQR) for continuous variables and as absolute numbers or percentages for categorical variables (sex, ASA PS etc.). Non-parametric and categorical variables were compared between survivors and non-survivors by Mann-Whitney *U* test, and binary variables were compared by Fisher exact test. Receiver-operating characteristic (ROC) curves were constructed for all baseline scores (such as SOFA, qSOFA and MPI), lactate levels at all study points (preoperative, at the end of surgery and at 24 h) and lactate clearance (lactate clearance at the end of surgery and 24 h) as predictors of 28-day mortality, and best cut off values were obtained from Youden’s index (= sensitivity + specificity- 1, )[14]. Area under the ROC curves with 95% confidence interval (95% CI) were reported for all variables.

Joint predictive ability of MPI and lactate clearance for 28-day mortality was tested by generalised linear model of binomial family, and the best model was chosen by lowest Akaike information criteria (AIC). Model fit was checked by Hosmer-Lemeshow goodness-of-fit statistics.

As a complementary method of logistic regression, a decision tree model was constructed by recursive partitioning method to identify most important variables to predict 28-day mortality. All analyses were conducted in R with pROC, Epi & rpart packages (R version 3.6.1, R Development Core Team, 2010; R Foundation for Statistical Computing, Vienna, Austria).

## Results

In this prospective study, *n* = 113 patients were recruited with a median (IQR) age of 32 (23, 48) years and 73 were male (64.6%). One hundred and two patients were ASA PS I and rest of them were ASA II. Within the follow-up period of 28 days, 17 patients [proportion (95% CI) 15.04 (9.6–22.8)] died. Median (IQR) length of hospital stay was 8 (6, 15) days and median (IQR) length of ICU stay was 0 (0, 2) days. All the baseline demographics of survivors (*n* = 96) and non survivors (*n* = 17) are listed in Table 1. Incidence [proportion (95% CI)] of acute kidney injury, need for vasopressor requirement and need for mechanical ventilation were [6.1% (2.9, 12.0)], [27.4% (20.3, 6.3)] and [30.1% (22.4–39.1)] respectively.

**Table 1:** Baseline demographics and disease severity predicting scores in all patients [Data presented as median (IQR) or proportion, as applicable]

Parameters	All patients (*n* = 113)	Survivors (*n* = 96)	Non survivors (*n* = 17)	Significance
Age (years)	32 (23, 48)	22 (19, 30.5)	49 (28, 58)	*p* = 0.021
Gender (male/female)	73/40	61/35	12/5	*p* = 0.784
SOFA	1 (1, 2)	1 (1, 1)	2 (1, 3)	*p* = 0.004
APACHE II	3 (2, 4)	2 (1, 3)	6 (5, 6)	*p* < 0.0001
qSOFA	1 (0, 1)	1 (1, 1)	1 (1, 1)	*p* = 0.00
MPI	11 (10, 16)	11 (9, 15)	17 (16, 22)	*p* = 0.00017
ASA PS (I/II)	102/11	92/4	10/7	*p* = 0.00
Preoperative lactate (mmol/l)	1.6 (0.9, 2.5)	1.35 (0.9, 2.1)	3.7 (2.8, 4.2)	*p* < 0.0001
Preoperative ScVO_2_	78 (65.25, 86.5)	82 (70.3, 87.6)	68 (64.7, 80.4)	*p* = 0.086
Site of perforation (A/B/C/D)	8/59/31/15	8/44/30/14	0/15/1/1	*p* = 0.016
AKI (yes/no)	7/106	2/94	5/12	*p* = 0.001
Need for vasopressor support (yes/no)	32/81	15/81	17/0	*p* < 0.001
Need for mechanical ventilation (yes/no)	34/79	17/79	17/0	*p* < 0.001

*IQR* interquartile range, *SOFA* Sequential Organ Failure Assessment score, *APACHE II* Acute Physiologic Assessment and Chronic Health Evaluation II, *qSOFA* Quick Sequential Organ Failure Assessment Score, *MPI* Mannheim Peritonitis Index, *ASA PS* American Society of Anesthesiologist Physical Status, *ScVO*_*2*_ Superior venacaval oxygen saturation, *A* stomach, *B* small intestine, *C* caecum with appendix, *D* colon, rectum and anal canal

.Non-survivors at 28 days were older [median (IQR) 49 years (28, 58) versus 22 years (19, 30.5); *p* = 0.021], had a longer ICU stay [median (IQR)] 6 (4, 9) days versus 0 (0, 1) days, *p* < 0.0001] but similar length of hospital stay [median (IQR)] 7 (5, 10) days versus 9 (6, 15) days, *p* = 0.195]. Preoperative SOFA score [median (IQR) 2 (1, 3) versus 1 (1, 2); *p* = 0.004], quick- SOFA score [median (IQR) 1 (1, 2) versus 1 (1, 1); *p* < 0.0001], APACHE II score [median (IQR)] 6 (5, 6) versus 2 (1, 3); *p* < 0.0001] and MPI score [median (IQR) 17 (16, 22) versus 11 (9, 15); *p* = 0.00017] were higher in non-survivors than survivors.

Preoperative [median (IQR) 3.7 (2.8, 4.2) mmol/l versus 1.35 (0.9, 2.1) mmol/l; *p* < 0.0001], postoperative [median (IQR) 3.6 (3.1, 4.2) mmol/l versus 1.4 (1, 2.2) mmol/l; *p* < 0.0001] and 24-h postoperative arterial lactate [median (IQR) 4.2 (3.7, 4.9) mmol/l versus 1.15 (0.9, 1.4) mmol/l; *p* < 0.0001] were higher in non survivors than survivors.

**Fig. 1:**
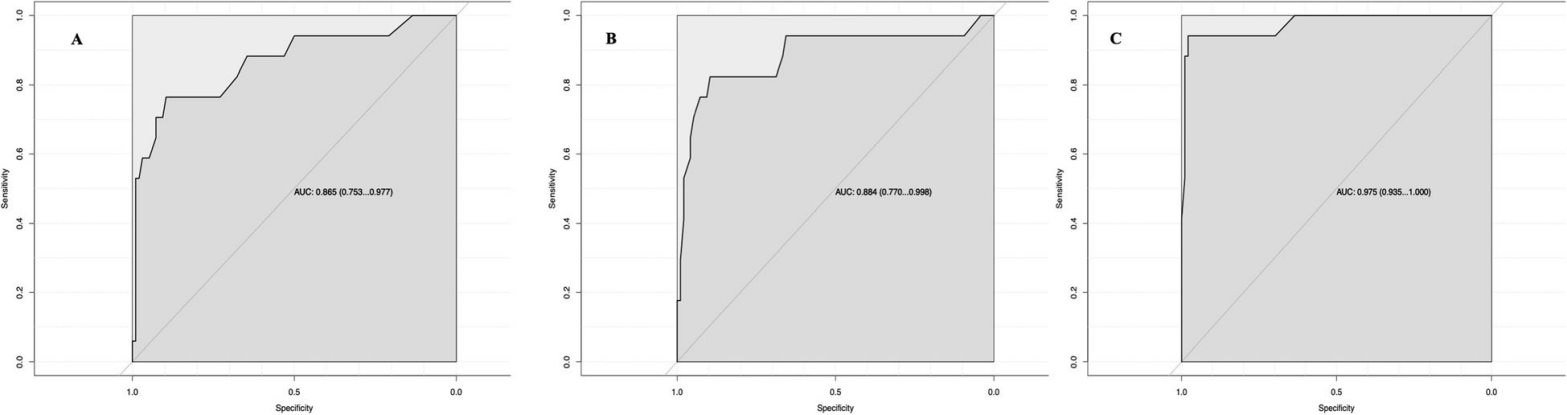
Area under the receiver operating characteristic curve of **a** preoperative arterial lactate, **b** arterial lactate after surgery, **c** arterial lactate 24 h after surgery to predict 28-day mortality

Receiver-operating characteristic (ROC) curves were constructed for preoperative lactate level, immediate postoperative lactate level, 24-h postoperative lactate level (Fig. 1) to predict 28-day mortality. The AUROC (95% CI) to predict 28-day mortality along with best cut off value and sensitivity and specificity for preoperative lactate, postoperative lactate, 24-h lactate are presented in Table 2.

**Table 2:** Prognostic performance of arterial lactate to predict 28-day mortality

Parameters	AUROC (95% CI)	Best cut off (mol/L)	Specificity	Sensitivity
Preoperative arterial lactate for 28-day mortality	0.865 (0.753–0.977)	2.75	89.58%	76.47%
Arterial lactate after surgery for 28-day mortality	0.884 (0.770–0.998)	2.8	89.58%	82.35%
Arterial lactate 24 h after surgery for 28-day mortality	0.975 (0.935–1.000)	2.45	97.92%	94.11%

The AUROC (95% CI) for MPI to predict 28-day mortality was 0.784 (0.637–0.931) and for APACHE II, it was 0.846 (0.743–0.948) (Table 3).

**Table 3:** Prognostic performance of various disease severity scores to predict 28-day mortality

Parameters	AUROC (95% CI)	Best cut off	Specificity	Sensitivity
MPI for 28-day mortality	0.784 (0.637–0.931)	> 16	88.54%	70.59%
Preoperative SOFA for 28-day mortality	0.698 (0.550–0.845)	> 2	94.79%	41.18%
Preoperative qSOFA for 28-day mortality	0.758 (0.632–0.885)	> 1	100%	47.05%
APACHE II for 28-day mortality	0.846 (0.743–0.948)	> 4	87.50%	76.47%

*AUROC* Area Under Receiver-Operating Characteristic Curve, *APACHE II* Acute Physiologic Assessment and Chronic Health Evaluation II, *qSOFA* Quick Sequential Organ Failure Assessment score, *MPI* Mannheim Peritonitis Index

A stepwise binary logistic regression model was consctructed with all variables associated with mortality at *p* < 0.05 and model with lowest Akaike information criteria (AIC) was selected. Stepwise regression revealed that a model consisting of MPI and 24-h post surgical lactate level was found to be the best predictor of 28-day mortality (Table 4).

**Table 4:** Prognostic performance of binary logistic regression models to predict 28 day mortality

Parameters	AUROC (95% CI)	Hosmer-Lemeshow goodness-of-fit	AIC
MPI + preoperative lactate	0.91 (0.85–0.98)	*X*^2^ = 4.20 *p* = 0.84	66.98
MPI + 24-h lactate	0.99 (0.98–1.00)	*X*^2^ = 4.25 *p* = 0.8	25.54

*MPI* Mannheim Peritonitis Index, *AUROC* Area Under Receiver Operating Characteristics Curve, *95% CI* 95% confidence interval, *AIC* Akaike information criteria

**Fig. 2:**
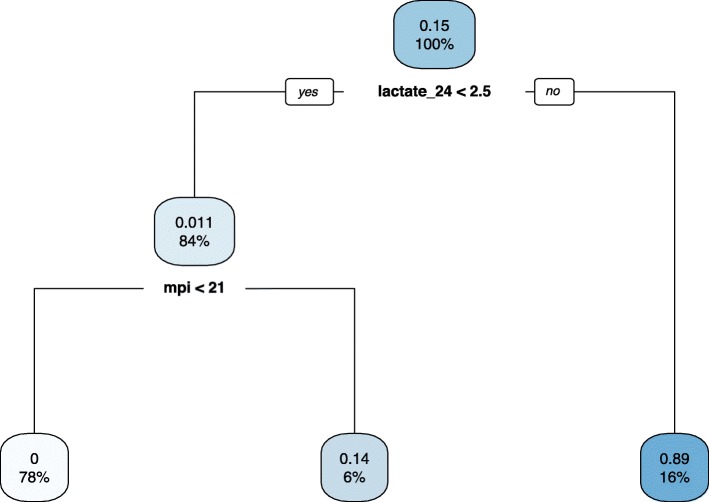
Decision tree model to predict 28-day mortality [24 h lactate was most informative variable followed by MPI]

We also used recursive partitioning and constructed decision tree to find out most informative predictor of 28-day mortality and found that 24-h lactate (at a cut off 2.5 mmol/l) was the most informative variable followed by MPI (cut off 21) [Fig. 2].

## Discussion

In this prospective observational study, preoperative, postoperative and 24-h postoperative arterial lactate were good predictors of 28-day mortality in patients undergoing emergency laparotomy for perforation peritonitis.

Lactic acid is an intermediate of carbohydrates and non-essential amino-acid metabolism, and its blood level is the net difference between its production and clearance. Lactic acid through Pasteur effect provides major energy [15, 16] to survive hypoxia. Lactate is produced in excess as a response to inflammatory mediators, as in peritonitis. Moreover, reduced lactate clearance may occur due to microcirculatory disarray, which could affect oxygen utilisation by mitochondria at the tissue level [17] and due to deranged renal function from sepsis or hypovolemia. Reversal of organ dysfunction in septic patients has been suggested to be part of a protective regulatory process, which induces a temporary hypometabolic state resembling hibernation that may protect the cells from dying and allow the possibility of functional recovery [18].

Both a single value of serum lactate and lactate clearance have been evaluated in various clinical scenarios as a prognostic marker. On analysis of the data from 28,150 patients using Surviving Sepsis Campaign database, an elevated serum lactate above 4 mmol/l with hypotension was found to be associated with mortality [19]. Another recent retrospective study on 2192 ICU patients with admission lactate more than 2.0 mmol/L, 24 h lactate clearance < 19% was associated with both increased in-hospital and long-term mortality, even after adjustment for APACHE II, need for catecholamines and intubation [20]. Lactate clearance at 6 h after admission in intensive care unit, was also found to be a predictor of mortality [21]. Similar findings were reported from a study conducted in patients admitted to surgical ICU also [22–24]. Higher lactate level was also associated with increased mortality and longer length of hospital stay in trauma patients [25].

However, lactate and lactate clearance at 24 h have been used to predict mortality predominantly in critically ill patients and not in surgical population [19–21]. For optimal prediction of prognosis by lactate clearance, one need to perform lactate value ideally every 1–2 hourly [26] or at least six times per day [20], which may not be feasible always. Moreover, the term lactate clearance may not be appropriate as it may sometimes reflect less overproduction and not elimination [26]. Therefore, preoperative, immediate postoperative and 24-h lactate value may be considered sufficient for predicting outcome, and use of lactate clearance can be avoided.

In the current study, 24-h postoperative lactate was the best predictor followed by preoperative and postoperative lactate values. So, 24-h postoperative lactate can be considered most important lactate value to predict mortality. It is important to note that by 24 h, patients were adequately resuscitated, appropriate antimicrobials started and adequate source control (laparotomy) was performed. So even after achieving these goals, hyperlactatemia remains a predictor of poor outcome.

However, initial resuscitation before and after surgery should be guided by baseline and subsequent lactate value, and persistent hyperlactatemia after resuscitation and surgery is predictor of poor outcome.

A single prospective study reported that though MPI was a predictor of mortality in perforation peritonitis patients, baseline hyperlactatemia was not a predictor [2]. However, with best of our knowledge, prognostic utility of serial lactate measurement in patients with perforation peritonitis has not been evaluated previously [10].

In our study MPI, preoperative lactate, intraoperative lactate and lactate 24 h after surgery are found to be independent predictors of mortality.

However, MPI needs intraoperative findings for calculation [11] which limits its use in preoperative assessment despite being easy to calculate. Addition of baseline serum lactate, 24-h postoperative lactate and 24-h lactate clearance with MPI score significantly increased its predictive accuracy for 28-day mortality, and MPI with 24-h lactate has an AUROC of 0.99. We, therefore, suggest the use of easily available lactate along with MPI score to predict severity of illness in peritonitis and thereby guiding intervention to improve patient outcome.

Most important strenght is that we have evaluated importance of serial lactate measurement along with other disease severity scores in patients with perforation peritonitis undergoing emergency laparotomy. However, our study has several limitations also. First, we recruited relatively small number of patients, and it was conducted in a single tertiary care center. Second, we could not validate our findings in an external cohort. Third, for optimal prediction of prognosis by lactate clearance, serial measurement of lactate should have been performed every 1–2 hourly [26] or at least six times per day [20] and we did not perform lactate so frequently.

## Conclusion

To conclude, preoperative, immediate postoperative and 24-h postoperative lactate value independently predicts 28-day mortality in perforation peritonitis patients undergoing emergency laparotomy. Combination of MPI and 24-h lactate value is the most accurate predictor of mortality.

## Data Availability

Available on request.

## Abbreviations

AIC: Akaike information criteria
APACHE II: Acute Physiology and Chronic Health Evaluation II
ASA PS: American Society of Anaesthesiologists Physical Status
AUROC: Area Under Receiver Operating Characteristic Curve
CVC: Central venous catheter
HDU: High dependency unit
KDIGO: Kidney Disease Improving Global Outcome
MAP: Mean arterial pressure
MODS: Multi organ dysfunction syndrome
MPI: Mannheim Peritonitis Index
NIBP: Non-invasive blood pressure
PACU: Post Anaesthesia Care Unit
PEEP: Positive end expiratory pressure
SOFA: Sequential Organ Failure Assessment score
